# Upregulation of Serum Sphingosine (d18:1)-1-P Potentially Contributes to Distinguish HCC Including AFP-Negative HCC From Cirrhosis

**DOI:** 10.3389/fonc.2020.01759

**Published:** 2020-09-08

**Authors:** Yingying Jiang, Cai Tie, Yang Wang, Dandan Bian, Mei Liu, Ting Wang, Yan Ren, Shuang Liu, Li Bai, Yu Chen, Zhongping Duan, Sujun Zheng, Jinlan Zhang

**Affiliations:** ^1^Difficult and Complicated Liver Diseases and Artificial Liver Center, Beijing Youan Hospital, Capital Medical University, Beijing, China; ^2^Beijing Municipal Key Laboratory of Liver Failure and Artificial Liver Treatment Research, Beijing Youan Hospital, Capital Medical University, Beijing, China; ^3^Institute of Materia Medica, Chinese Academy of Medical Sciences (CAMS) and Peking Union Medical College (PUMC), Beijing, China

**Keywords:** hepatocellular carcinoma, cirrhosis, AFP, sphingolipid, serum

## Abstract

**Background:**

Serum sphingolipids are widely involved in the development of hepatocellular carcinoma (HCC). We investigated the serum sphingolipid profile in patients with HCC or cirrhosis and explored the potential diagnostic efficiency of serum sphingolipid metabolites which may be helpful in differentiating HCC including α-fetoprotein (AFP)-negative HCC from cirrhosis.

**Methods:**

Seventy-two HCC patients (including 24 AFP-negative HCC) and 104 cirrhotic patients were consecutively enrolled in this study. High-performance liquid chromatography–tandem mass spectrometry was used to detect a panel of 57 serum sphingolipid metabolites.

**Results:**

Twenty-four sphingolipid metabolites showed significant differences between HCC and cirrhotic patients (all *P* < 0.05). Sphingosine (d18:1)-1-P was found to have the potential to differentiate HCC from cirrhosis by orthogonal partial least squares discriminant analysis (OPLS-DA). There was no significant difference in the efficacy of Sphingosine (d18:1)-1-P and AFP to distinguish HCC from cirrhosis, and the area under the receiver operating curve (AUC) were 0.85 and 0.83 (*P* > 0.05), respectively. When the cut-off value of Sphingosine (d18:1)-1-P was set at 56.29 pmol/0.1 ml, the sensitivity and specificity were 79.20% and 78.70%, respectively. Notably, the upregulation of Sphingosine (d18:1)-1-P could also distinguish AFP-negative HCC from cirrhosis with an AUC of 0.79. The sensitivity and specificity were 62.50% and 77.90% at a cut-off value of 56.29 pmol/0.1 ml. Spearman rank correlation analysis revealed that serum Sphingosine (d18:1)-1-P was not correlated with AFP in patients with cirrhosis, AFP-positive HCC, and AFP-negative HCC. Moreover, the difference in the diagnostic efficiency of serum Sphingosine (d18:1)-1-P was not statistically significant between tumor size (≤2 cm vs. >2 cm, *P* = 0.476). Also, there was no difference among patients with different TNM stages and BCLC stages.

**Conclusion:**

The upregulation of serum Sphingosine (d18:1)-1-P exhibits good diagnostic performance for HCC. Particularly, Sphingosine (d18:1)-1-P could also serve as a biomarker for the diagnosis of AFP-negative HCC. These findings may contribute to the non-invasive diagnosis of HCC including AFP-negative HCC.

## Introduction

Hepatocellular carcinoma (HCC) is the third leading cause of cancer-related deaths around the world, and 70–90% of HCC may evolve from patients with cirrhosis (1–4). Early discriminating HCC from cirrhosis and further providing timely treatment can significantly improve the prognosis of HCC. However, typical symptoms are absent in the early stage of HCC, and specific diagnostic biomarkers for HCC are deficient. Therefore, many patients are at an advanced stage when they were diagnosed. This fact makes many patients miss the best opportunity for curative therapy options, such as liver resection or liver transplantation (5, 6). Up to now, the imaging methods and α-fetoprotein (AFP) are commonly used to screen and diagnose HCC in clinical practice. Unfortunately, on the one hand, ultrasound does not provide sufficient sensitivity and objectivity which may lead to the heterogeneity of diagnosis; CT and MRI are not recommended as common screening tools for HCC because of the accompanying radiation exposure or high cost. On the other hand, the positive rate of AFP is limited to only one half to two thirds, namely, there is approximately 40% of HCC that cannot be detected (7–9). In addition, AFP can be affected by many non-HCC diseases (such as hepatitis, cirrhosis, cholangiocarcinoma, and so on). Therefore, there is still an urgent need for the discovery of novel biomarkers in the screening of HCC especially for AFP-negative HCC (10, 11).

**FIGURE 1:**
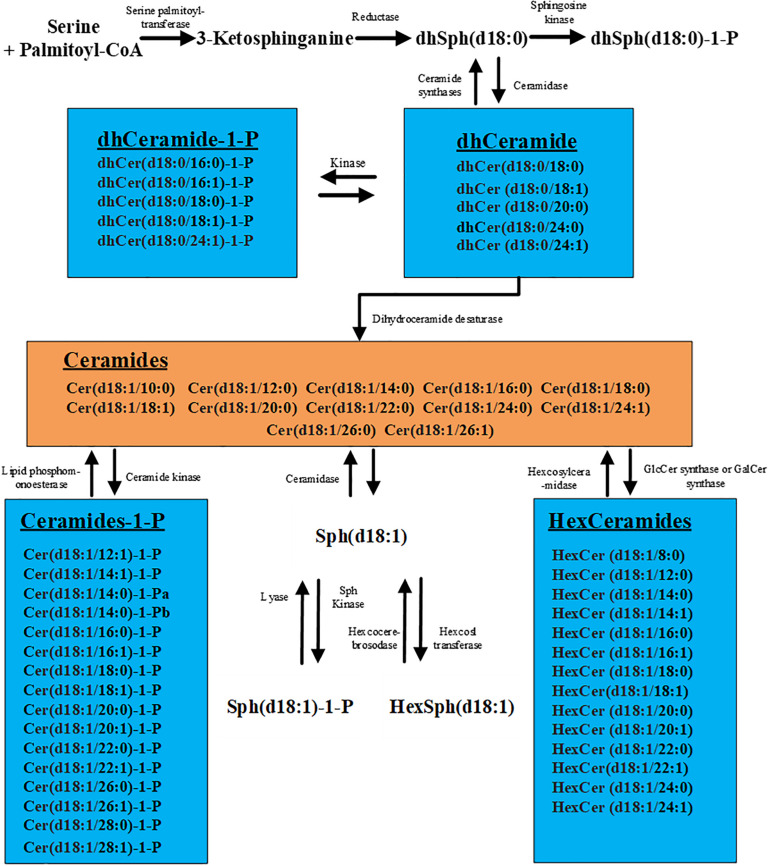
Metabolic pathways of sphingolipids. The figure contains all the 57 sphingolipids examined in this study.

High-performance liquid chromatography–tandem mass spectrometry (HPLC-MS/MS) has turned out to be a powerful tool for analyzing sphingolipid metabolites highly sensitively and specifically with the rapid progress of this technology (12). Sphingolipid metabolism is highly interconnected. As the core of sphingolipid metabolism network, ceramide can be glucosylated, phosphorylated, or deacylated to produce a wide array of metabolites (Figure 1). Recently, an increasing number of studies have shown that serum sphingolipid metabolites are not only the cellular structural components and the messenger molecules that regulate hepatocyte apoptosis, proliferation, and cell–cell interactions (13–17) but also were widely involved in the development of HCC (16, 18–20). A previous study reported that long-chain (C16–C20) and very long-chain (C22–C24) ceramides, dihydroceramides, sphingosine, and sphingosine-1-phosphate (S1P) are obviously upregulated in the serum of HCC patients compared with patients with cirrhosis (21). In another study, Cowart et al. (22) reported that S1P levels are reduced in HCC tissues compared with adjacent non-HCC liver tissues, and the downregulation of S1P in HCC tissues is associated with earlier relapse of HCC. These encouraging results indicate the key role of sphingolipids in HCC pathogenesis and also point the way for the discovery of new diagnostic markers. However, the species and amounts of sphingolipids detected in these studies were not comprehensive, such as some lengths (8, 10, 12, 14, 23, 24) of carbon chain of ceramide or S1P were not included, which might result in the miss of the optimal diagnostic biomarkers. Meanwhile, the sensitivity and specificity of these individual markers were not shown, and their diagnostic role for AFP-negative HCC was also not evaluated. Moreover, in view of the etiology heterogeneity and different ethnics, further screening sphingolipid metabolites which can distinguish HCC (including AFP-negative HCC) from cirrhosis with high sensitivity and specificity is imperative.

In this study, we used HPLC-MS/MS to quantify a panel of 57 serum sphingolipid metabolites in Chinese patients with HCC or cirrhosis, intending to explore the expression profile of serum sphingolipid metabolites in Chinese patients with HCC and to seek out the specific and sensitive sphingolipids which help differentiate HCC, including AFP-negative HCC, from cirrhosis.

## Materials and Methods

### Patient Population and Clinicopathological Data Collection

One hundred and seventy-six patients were consecutively enrolled in the present study from July 2014 to May 2015 in Difficult and Complicated Liver Disease and Artificial Liver Center, Beijing Youan Hospital, Capital Medical University, China. HCC was diagnosed according to the guidelines for diagnosis and treatment of primary liver cancer in China and EASL-EORTC (1, 25). Cirrhosis was diagnosed according to the established criteria (26), mainly based on the clinical data of imaging by ultrasonography, CT, MRI, the impaired liver function, and (or) histological evidence. Before blood sampling, none of the HCC patients received local or systemic treatment for HCC, which aimed to eliminate the impact of treatment on sphingolipid metabolites. Exclusion criteria were as follows: coexisting other serious active physical and mental diseases, including uncontrolled primary kidney, heart, lung, vascular, neurological, and metabolic diseases (hyperthyroidism, severe diabetes, and adrenal diseases); lipid metabolic abnormalities – severe hyperlipidemia, lipid storage disease, obesity (body mass index ≥25 kg/m^2^), immunodeficiency disease, or other systemic tumors; pregnancy or lactation.

Tumor size was defined as the maximum diameter of the lump for single lesion or the sum of two largest tumors’ diameter for multiple lesions based on the histopathologic features or medical imaging. In this study, patients were divided into two groups according to the tumor size (tumor size ≤2 and >2 cm). The TNM stage was defined according to the American Joint Committee on Cancer TNM Staging system for Liver Tumors as follows (27): primary tumor (T): (TX) Primary tumor cannot be assessed; (T0) No evidence of primary tumor; (T1) Solitary tumor without vascular invasion; (T2) Solitary tumor with vascular invasion or multiple tumors, none more than 5 cm in greatest dimension; (T3) Multiple tumors any more than 5 cm or tumor involving a major branch of the portal or hepatic vein(s); (T4) Tumor(s) with direct invasion of adjacent organs, other than the gallbladder or with perforation of visceral peritoneum. Regional lymph nodes (N): (NX) Regional lymph nodes cannot be assessed; (N0) No regional lymph node metastasis; (N1) Regional lymph node metastasis. Distant metastasis (M): (M0) No distant metastasis; (M1) Distant metastasis. The TNM staging system can be found at Supplementary Table S1. Early stage (A), intermediate stage (B), advanced stage (C), and end-stage disease (D) were classificated according to the Barcelona clinic liver cancer (BCLC) staging system (23).

The study was conducted in compliance with the Declaration of Helsinki. The research protocol was approved by the Medical Ethics Review Committee of Beijing Youan Hospital, Capital Medical University. All participants provided written consent.

### Biochemistry Detection, Serum AFP Quantification, and Scoring Systems

Alanine aminotransferase (ALT) and aspartate aminotransferase (AST) were measured using an Olympus Automatic Biochemical Analyzer AU5400 (Olympus, Tokyo, Japan). The AFP level was determined using an automatic electrochemiluminescence immunoassay (Roche Diagnostics, Shanghai, China). AFP negative was defined as the serum concentration below 20 ng/ml.

Various non-invasive markers, such as model for end-stage liver disease (MELD), fibrosis 4 index (FIB-4), and aspartate aminotransferase to platelet ratio index (APRI) (24, 28), were assessed in this study, which were calculated according to clinical and laboratory parameters at the time of inclusion. The formulas for calculating MELD, APRI, and FIB-4 scores are as follows:

$$\begin{array}{l} MELD=3.8\times \ln(TBIL)+11.2\times \ln(INR)+9.6\times \ln(Cr)\\ \;+6.4\times Etiology(Bilious\;or\;alcoholic)=0,\\ \;others=1) \end{array}$$

$$\begin{array}{l} FIB-4=\frac{age\times AST}{PLTcount\times \sqrt{ALT}}\\ \;APRI=\frac{AST}{upperlimitofnormal\times PLT}\times 100 \end{array}$$

### Determination of Serum Sphingolipid Metabolites

The determination of serum sphingolipid metabolites using the HPLC-MS/MS was performed as previously described (10, 15, 18, 29). HPLC-MS/MS was performed using an Agilent 6410B Triple Quad mass spectrometer (Agilent Technologies, Santa Clara, CA, United States) comprising a triple quadrupole MS analyzer equipped with an electrospray ionization interface and an Agilent 1200 RRLC system (HPLC-MS/MS).

### Statistical Analysis

Data were analyzed using the IBM SPSS statistical software package 22.0 (SPSS, Chicago, IL, United States). Unless otherwise specified, *P* < 0.05 were considered to be statistically significant in two-tailed test. Continuous variables were expressed as mean ± SD or median (range) depending on whether the data obeyed normal distribution or not. Dichotomy variables were expressed as number or percentage. Univariate analysis was performed according to data characteristics, continuous quantitative data were compared between the two groups using *t* test or Mann–Whitney *U* test. The comparison between the counting data sets is performed using a χ^2^ test. Multivariate logistic regression analysis (forward stepwise method) was performed to seek out the sphingolipid metabolites that were independently associated with total HCC or AFP-negative HCC, and the *P* values of entry and removal were set to 0.05 and 0.1, respectively. Correlation analysis was performed using Spearman rank test. Kruskal–Wallis test was used among multiple independent samples. The diagnostic performance of potential serum biomarker was calculated by the area under the receiver operating characteristic (ROC). The AUC difference is performed using DeLong’s test.

Orthogonal partial least squares discriminant analysis (OPLS-DA), a multivariate analysis, was used to visually discriminate between HCC or AFP-negative HCC and cirrhotic patients using SIMCA 13.0 software (Umetrics, Umeå, Sweden). Sphingolipids data were mean centered and UV scaled. The quality of each OPLS-DA model was examined using the R2Y (cum) and Q^2^ (cum) values, which are used to assess the stability and predictability of the model, respectively. The criteria for the selection of potential biomarkers were as follows: the value of the variable importance in projection was greater than 1; the jack-knife uncertainty bar excluded zero; the absolute value of *P-corr* in the *S-plot* was 0.58. The cross-validation parameter, Q^2^, was calculated as in partial least squares discriminant analysis by permutation testing using 100 random permutations to test the validity of the model against over-fitting.

## Results

### Demographic and Clinical Characteristics of Patients

A total of 72 HCC patients, including 24 serum AFP-negative HCC patients, and 104 patients with cirrhosis were included in the present analysis. Among these groups, the difference of gender, age, and liver functions (including ALT, AST, TBIL, DBIL, TP, and ALB) was not significant (Table 1). The MELD scores in cirrhotic patients were remarkably higher than those in patients with HCC. This could be easily understood by the fact that patients with cirrhosis often seek medical treatment because of various complications, but the HCC patients enrolled in our study were diagnosed for the first time. There were 75 (72.12%) and 49 (68.06%) male patients in cirrhosis and HCC group with a mean age of 54.26 and 56.28 years, respectively. The etiology of cirrhosis and HCC patients mainly includes HBV (47.12 vs. 58.33%), HCV (14.42 vs. 6.94%), alcoholic liver disease (16.34 vs. 4.17%), and others (22.12 vs. 30.56%). The detailed demographic and clinical characteristics of the participants are shown in Table 1.

**TABLE 1:** Demographic and clinical characteristics of patients.

Variables	Cirrhosis (*N* = 104)	HCC (*N* = 72)	*P*^†^	AFP-negative HCC (*N* = 24)	*P*^‡^
Age (years), means ± SD	54.26 ± 11.64	56.28 ± 10.93	0.25^††^	58.50 ± 11.78	0.24^††^
Male (sex), n (%)	75.00(72.12)	49(68.06)	0.37^‡‡^	18.00(75.00)	0.78^‡‡^
AFP (ng/ml), median (range)	3.7 (0.79–592.20)	56.99 (0.92–121000.00)	<0.01**	5.38 (0.92–15.29)	0.46**
ALT (U/L), median (range)	28.25 (3.90–1048.50)	36.15 (9.70–269.20)	0.05**	38.10 (13.60–172.90)	0.18**
AST (U/L), median (range)	40.15 (16.90–350.70)	52.45 (13.40–339.30)	0.15**	49.30 (21.70–235.80)	0.32**
TBIL (μmol/L), median (range)	28.1 (9.60–373.80)	23.35 (6.20–437.70)	0.11**	24.55 (9.70–437.70)	0.49**
DBIL (mol/L), median (range)	7.35 (1.5–200.03)	6.60 (0.30–227.80)	0.54**	6.65 (2.10–227.80)	0.68**
TP (g/L) means ± SD	61.29 ± 8.60	61.75 ± 6.12	0.90^††^	60.98 ± 6.22	0.72^††^
ALB (g/L) means ± SD	34.07 ± 5.95	35.57 ± 5.18	0.10^††^	36.49 ± 5.16	0.06^††^
MELD, median (range)	10 (6.00–22.00)	9.00 (6.00–25.00)	0.04**	10.00 (6.00–19.00)	0.85**
FIB-4, median (range)	5.30 (1.09–52.97)	5.28 (0.52–30.55)	0.41**	5.22 (1.10–15.84)	0.69**
APRI, median (range)	1.45 (0.2–12.38)	1.50 (0.12–9.20)	0.58**	1.52 (0.12–9.20)	0.81**
Etiology					
HBV, n (%)	49 (47.12)	42 (58.33)	0.14^‡‡^	10 (41.67)	0.63^‡‡^
HCV, n (%)	15 (14.42)	5 (6.94)	0.12^‡‡^	2 (8.33)	0.65^‡‡^
ALD, n (%)	17 (16.34)	3 (4.17)	0.01^‡‡^	2 (8.33)	0.50^‡‡^
Other*, n (%)	23 (22.12)	22 (30.56)	0.21^‡‡^	10 (41.67)	0.04^‡‡^

Data are expressed as means ± SD, median (range) or n (%). ^††^Indicates the comparison between cirrhosis and HCC or AFP-negative HCC using *t* test. ^‡‡^Indicates the comparison between cirrhosis and HCC or AFP-negative HCC using χ^2^ test. **Indicates the comparison between cirrhosis and HCC or AFP-negative HCC using Mann–Whitney *U* test. *P*^†^ indicates the comparison between cirrhosis and HCC. *P*^‡^ indicates the comparison between cirrhosis and AFP-negative HCC. *P* < 0.05 is considered to be statistically significant. *Other etiology including primary biliary cirrhosis (PBC), cryptogenic, hepatic veno-occlusive disease (VOD), liver disease with two or more causes. HCC, hepatocellular carcinoma; AFP, α-Fetoprotein; ALT, alanine aminotransferase; AST, aspartate aminotransferase; TBIL, total bilirubin; DBIL, direct bilirubin; TP, total protein; ALB, albumin; MELD, model for end-stage liver disease; FIB-4, Fibrosis 4 Score; APRI, Aspartate aminotransferase-to-Platelet Ratio Index; ALD, alcoholic liver disease.

Further, we counted the number of HCC patients with distant metastasis, intrahepatic metastasis, and portal vein invasion. Of note, the data of tumor size and metastasis status were missed in two AFP-positive HCC patients, so these two patients were excluded from the tumor metastasis analysis. The result showed that AFP-negative HCC patients had a lower incidence of distant metastasis (*P* < 0.001, χ^2^ test), intrahepatic metastasis (*P* = 0.007, χ^2^ test), and portal vein invasion (*P* = 0.016, χ^2^ test) compared with AFP-positive HCC patients. The detailed information are shown in Table 2.

**TABLE 2:** Tumor metastasis in AFP-negative HCC and AFP-positive HCC patients.

	Distant metastasis			Intrahepatic metastasis			Portal vein invasion		
	Yes (*N* = 41)	No (*N* = 29)	*P*^‡‡^	Yes (*N* = 20)	No (*N* = 50)	*P*^‡‡^	Yes (*N* = 25)	No (*N* = 45)	*P*^‡‡^
AFP-negative HCC (*N* = 24)	3	21	–	2	22	–	4	20	–
AFP-positive HCC (*N* = 46)*	38	8	<0.001	18	28	0.007	21	25	0.016

*Two AFP-positive HCC patients were excluded from the analysis due to the missing data of distant metastasis, intrahepatic metastasis, and portal vein invasion. ^‡‡^Indicates the comparison between AFP-positive or AFP-negative HCC using χ^2^ test.

### Serum Sphingolipid Profile in Patients With HCC or Cirrhosis

**FIGURE 2:**
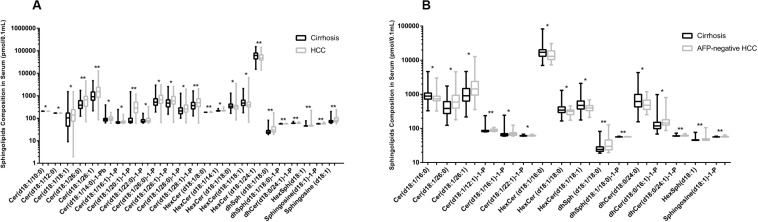
Comparison of serum sphingolipid levels (only the serum sphingolipids with significant difference are shown). **(A)** Twenty-four sphingolipid metabolites were significantly different between HCC (*n* = 72) and cirrhotic patients (*n* = 104). **(B)** Sixteen sphingolipid metabolites were significantly different between AFP-negative HCC (*n* = 24) and cirrhotic patients (*n* = 104). Data are expressed as median (range) (*P < 0.05, **P < 0.01, Mann–Whitney *U* test).

**FIGURE 3:**
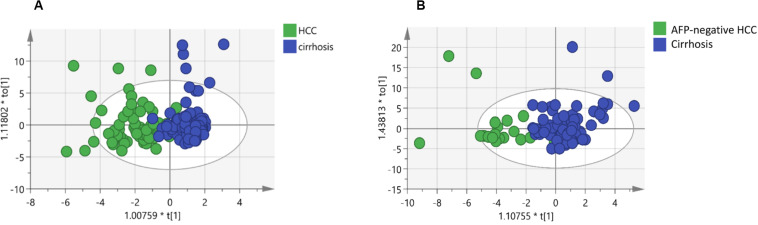
Identification of potential biomarkers through OPLS-DA: **(A)** Score plots obtained from the cirrhosis (*n* = 104) and HCC patients (*n* = 72). **(B)** Score plots obtained from the cirrhosis (*n* = 104) and AFP-negative HCC (*n* = 24) patients. Different colors of these dots represent different groups which are well separated based on sphingolipid levels. Also, the data indicate serum sphingolipidome varies significantly among groups. The scatter plot of t[1] vs to[1] is a window in the X space in which the separation of the two classes of observations occurs in the horizontal (t1) direction. The vertical (to[1]) direction expresses within class variability. * means t[1] or to[1] multiply by a coefficient, all these were automatically generated by the SIMCA software.

**FIGURE 4:**
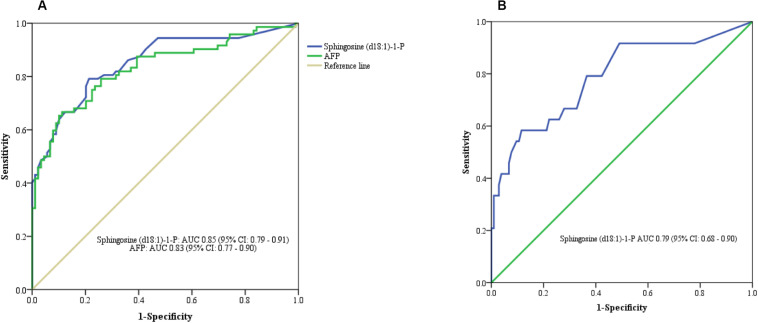
ROC curve analysis. **(A)** Serum Sphingosine (d18:1)-1-P for differentiating HCC from cirrhotic patients. **(B)** Serum Sphingosine (d18:1)-1-P for differentiating APF-negative HCC from cirrhotic patients.

In this study, we quantified a panel of 57 serum sphingolipid metabolites in Chinese patients with HCC (*n* = 72) or cirrhosis (*n* = 104). Twenty-four sphingolipid metabolites were significantly different between HCC and cirrhosis patients (*P* < 0.05, Mann–Whitney *U* test), including three downregulated (1 Cer, 1 C1P, 1 HexCer) and 21 upregulated (4 Cer, 1 dhSph, 1 dhS1P, 1 dhC1P, 4 HexCer, 7 C1P, 1 Sph, 1 S1P, 1 HexSph) sphingolipid metabolites in HCC patients (Figure 2A). The detailed characteristics of the 57 serum sphingolipid profile in patients with HCC or cirrhosis are shown in Supplementary Table S2. OPLS-DA results revealed that Sphingosine (d18:1)-1-P was the potential metabolite that could differentiate HCC from cirrhosis; the R2Y (cum) and Q^2^ (cum) were 0.717 and 0.604, respectively (Figure 3A). The diagnostic performance of this serum sphingolipid was assessed by ROC analysis, and the AUC was 0.85 (95% CI 0.79–0.91) (*P* < 0.001). When the cut-off value was set at 56.29 pmol/0.1 ml, the sensitivity and specificity were 79.20% and 78.70%, respectively. To compare the diagnostic value of Sphingosine (d18:1)-1-P and AFP, we also evaluated the AUC of AFP, which was 0.83 (95% CI 0.77–0.90) (*P* < 0.001). When the cut-off value was set at 20.32 ng/ml, the sensitivity and specificity were 66.70% and 88.80%, respectively. Though the AUC of Sphingosine (d18:1)-1-P was higher than that of AFP, the difference was not significant (*P* > 0.05, DeLong’s test) (Figure 4A).

### Serum Sphingolipid Profile in Patients With AFP-Negative HCC or Cirrhosis

The difference in baseline clinical parameters was not significant between AFP-negative HCC and cirrhosis (Table 1). Sixteen sphingolipid metabolites exhibited obvious difference (*P* < 0.05, Mann–Whitney *U* test), including five downregulated (1 Cer, 3 HexCer, 1 dhCer) and 11 upregulated (2 Cer, 3 C1P, 2 dhC1P, 1 dhSph, 1 dhS1P, 1 HexSph, 1 S1P) sphingolipids (Figure 2B). The detailed information of the 57 serum sphingolipid profiles in patients with AFP-negative HCC is also shown in Supplementary Table S2.

Orthogonal partial least squares discriminant analysis was performed to discriminate the AFP-negative HCC from cirrhosis. The result indicated that Sphingosine (d18:1)-1-P can also differentiate AFP-negative HCC from cirrhosis patients, and the R2Y (cum) and Q^2^ (cum) were 0.568 and 0.464, respectively (Figure 3B). The AUC of Sphingosine (d18:1)-1-P was 0.79 (95% CI 0.68–0.90) (*P* < 0.001), and the sensitivity and specificity were 62.50% and 77.90%, respectively, when the cut-off value was set at 56.29 pmol/0.1 ml (Figure 4B).

### Correlation Between Serum Sphingosine (d18:1)-1-P and Clinical Parameters

The level of AFP in cirrhotic patients was remarkably lower than that in HCC (*P* < 0.01, Mann–Whitney *U* test), whereas the difference of AFP level between cirrhosis and AFP-negative HCC was not significant (*P* > 0.05, Mann–Whitney *U* test). Spearman rank correlation analysis revealed that the level of serum Sphingosine (d18:1)-1-P was not correlated with AFP in cirrhosis (*r* = −0.04, *P* = 0.71), total HCC (*r* = 0.06, *P* = 0.60), AFP-positive HCC (*r* = −0.09, *P* = 0.56), or AFP-negative HCC (*r* = −0.13, *P* = 0.55), respectively.

To explore the relationship between Sphingosine (d18:1)-1-P and tumor size, we divided HCC patients into two groups according to tumor size (tumor size ≤2 cm and >2 cm). As mentioned earlier, two AFP-positive HCC patients, whose data of tumor size and metastasis status were missed, were excluded from the following analysis. No statistically significant difference was found in the level of serum Sphingosine (d18:1)-1-P between these two groups (58.09 ± 3.09 vs. 58.11 ± 2.54 pmol/0.1 ml, *P* = 0.476, Mann–Whitney *U* test). In addition, we divided HCC patients into four groups according to the TNM staging system, namely TNM I, TNM II, TNM III, and TNM IV. The level of serum Sphingosine (d18:1)-1-P was 58.18 ± 2.91, 57.78 ± 1.57, 58.74 ± 3.15, and 58.20 ± 3.31 pmol/0.1 ml, respectively. There was no difference among these groups (*P* = 0.771, Kruskal–Wallis test). Furthermore, the difference between any two groups was also not significant (all *P* > 0.05, Mann–Whitney *U* test). We then divided HCC patients into four groups according to the BCLC staging systems, namely, A, B, C, and D. The level of serum Sphingosine (d18:1)-1-P was 58.45 ± 2.81, 57.91 ± 1.62, 59.10 ± 3.50, and 58.30 ± 3.15 pmol/0.1 ml, respectively. Likewise, the difference between any two groups was not significant (all *P* > 0.05, Mann–Whitney *U* test).

## Discussion

Currently, the identification of novel serum biomarkers for the diagnosis of HCC, including AFP-negative HCC, is still less than desirable. Studies have found that compared with AFP-positive HCC patients, AFP-negative HCC patients were less likely to feature cirrhosis nodules and poor Edmondson–Steiner grade; conversely, they are more likely to form complete tumor capsules and have a favorable long-term prognosis (30–33). Our study also found that AFP-negative HCC patients have a lower incidence of distant metastasis, intrahepatic metastasis, and portal vein invasion compared with AFP-positive HCC patients. Therefore, it is vital to identify these patients and provide timely treatment, so as to improve survival rate and prognosis of AFP-negative HCC patients. This study reported that serum Sphingosine (d18:1)-1-P can potentially differentiate HCC, including AFP-negative HCC from cirrhosis. To the best of our knowledge, this is the first report on the potential capability of Sphingosine (d18:1)-1-P to effectively discriminate AFP-negative HCC from cirrhosis.

A variety of studies have revealed that sphingolipids may participate in the pathogenesis of liver disease (34, 35). However, the ability of serum sphingolipids to screen or diagnose HCC patients remains an issue to be further explored. In this study, we found that 10 sphingolipids (2 Cer, 1 C1P, 2 HexCer, 1 dhSph, 1 dhS1P, 1 dhC1P, 1 HexSph, 1 S1P) including Sphingosine (d18:1)-1-P exhibit significant difference between HCC or AFP-negative HCC and cirrhosis. Except for two HexCer, the remaining eight sphingolipids are upregulated in patients with AFP-negative HCC, and most of them belong to long-chain or very long-chain sphingolipids. Although the underlying mechanism was unclear, our finding lays a preliminary foundation for further study on the pathogenesis of HCC and AFP-negative HCC.

Grammatikos and colleagues observed that ceramides are significantly upregulated in the serum of patients with HCC. Meanwhile, their upstream synthetic precursor dihydroceramides and the downstream products including sphingosine and S1P are also upregulated in HCC patients. Furthermore, ROC analysis revealed C16 ceramide and S1P show the highest AUC (0.999 and 0.985, respectively) (21). Nevertheless, this study did not report the relationship between AFP-negative HCC and sphingolipids. Our findings are consistent with this report in some respects. Both studies showed that most of the ceramides are upregulated significantly in HCC patients, and the downstream product Sphingosine (d18:1)-1-P is also markedly upregulated. In addition, most of these sphingolipids belong to long-chain (C16–C20) and very long-chain (≥C22) sphingolipids. Of note, compared with Grammatikos’ study, we have detected sphingolipids with more types and numbers, which provides more chance and more convinced evidence for seeking out the optimal diagnostic biomarkers. Moreover, we found that the upregulation of Sphingosine (d18:1)-1-P is the most closely related to HCC, other than C16 ceramide and S1P reported in the study of Georgios et al. This difference might be explained by the diversity of etiology, region, ethnicity, and so on. Georgios’ study involves German patients, and the etiology of cirrhosis is mainly ascribed to alcoholic abuse (54.3%) and HCC was mainly caused by HCV (32.7%) or HBV (18.8%). This is distinct from the common etiology of cirrhosis and HCC in China where both cirrhosis (47.12%) and HCC (58.33%) are mainly caused by HBV.

Ceramide stands at the core of sphingolipid metabolism network. Sphingosine (Sph) can be generated under the action of ceramase and further be phosphorylated into S1P by Sphingosine kinase 1 (SphK1) (Figure 1). Cumulative evidences have demonstrated that S1P can promote tumor proliferation, migration, transformation, and participate in the growth and invasion of cancer cells (36–38). Cheng et al. (39) showed that S1P could act as an upstream repressor of Hippo pathway, which induces the activation of YAP in HCC cells and leads to the occurrence of HCC. In addition, inhibiting the activation of sphingosine kinase could promote the apoptosis of HCC cells (40). In our study, Sphingosine (d18:1)-1-P is discovered to possess the capacity to distinguish patients with HCC from cirrhosis. A previous study demonstrated that upregulated Sphingosine (d18:1)-1-P involves in the pathogenesis of HCC, which can be easily inferred from the fact that S1P derives from ceramide and it has been shown to be involved in cancer cell growth and invasion (41). Interestingly and importantly, our findings firstly suggest Sphingosine (d18:1)-1-P can effectively discriminate AFP-negative HCC from cirrhosis. In addition, we found that the level of Sphingosine (d18:1)-1-P is not correlated with AFP concentration in patients with cirrhosis or HCC, suggesting that the regulatory mechanism of Sphingosine (d18:1)-1-P in HCC patients may be independent of AFP. However, whether some other intermediate sphingolipids that are involved in the expression of AFP remains to be elucidated. Our results also revealed that the difference in the level of serum Sphingosine (d18:1)-1-P is not statistically significant between the two groups (tumor size ≤2 cm or >2 cm). Likewise, there was no difference among patients with different TNM stages and BCLC stages. Therefore, it can be inferred from these results that Sphingosine (d18:1)-1-P can be applied to identify all kinds of HCC patients, regardless of tumor size, TNM stage, and BCLC stage.

Although our results are promising, there are limitations. First, the clinical correlation study cannot be used to clarify the causal relationship between sphingolipid metabolites and HCC. Second, this is a cross-sectional study, thus the dynamic change of Sphingosine (d18:1)-1-P in response to HCC treatment is unable to be displayed. Third, the insufficient case number of HCC patients might impair the persuasive power of our findings. Collectively, whether this sphingolipid metabolite can be used to predict the prognosis of HCC patients still needs further investigation.

In conclusion, upregulation of serum Sphingosine (d18:1)-1-P may potentially help to differentiate HCC, including AFP-negative HCC, from cirrhosis. In the future, *in vitro* and *in vivo* studies that shed light into the pathophysiologic links between sphingolipid metabolites and HCC will contribute to the development of novel therapeutic strategies.

## Data Availability Statement

All datasets generated for this study are included in the article/Supplementary Material.

## Ethics Statement

The studies involving human participants were reviewed and approved by the research protocol was approved by the Medical Ethics Review Committee of Beijing Youan Hospital, Capital Medical University. The patients/participants provided their written informed consent to participate in this study.

## Author Contributions

SZ and JZ contributed to the conception and design of the study. YJ, CT, YW, DB, ML, TW, YR, SL, LB, YC, and ZD organized the database. JZ contributed to the ideas on method application. CT performed the sphingolipid detection. YJ and YW analyzed the data and wrote the manuscript. All authors contributed to article revision, and read and approved the submitted version.

## Conflict of Interest

The authors declare that the research was conducted in the absence of any commercial or financial relationships that could be construed as a potential conflict of interest.
