# Supplementary material for: Upregulation of Serum Sphingosine (d18:1)-1-P Potentially Contributes to Distinguish HCC Including AFP-Negative HCC From Cirrhosis
Source: Front Oncol. 2020 Sep 8;10:1759. doi: 10.3389/fonc.2020.01759 (PMC7506152; doi:10.3389/fonc.2020.01759)
Supplement: Supplementary file 1 [file Data_Sheet_1.docx]

**SUPPLEMENTARY TABLE 1 |** The American Joint Committee on Cancer Classiﬁcation

| **T – Primary tumor** |
| --- |
| TX Primary tumor cannot be assessed. |
| T0 No evidence of primary tumor. |
| T1 Solitary tumor without vascular invasion. |
| T2 Solitary tumor with vascular invasion or multiple tumors, none more than 5 cm in greatest dimension. |
| T3 Multiple tumors any more than 5 cm or tumor involving a major branch of the portal or hepatic vein(s). |
| T3a: Multiple tumors any more than 5 cm. |
| T3b: Tumors involving a major branch of the portal or hepatic vein(s). |
| T4 Tumor(s) with direct invasion of adjacent organs other than the gallbladder or with perforation of visceral peritoneum. |
| **N – Regional lymph nodes** |
| NX Regional lymph nodes cannot be assessed. |
| N0 No regional lymph node metastasis. |
| N1 Regional lymph node metastasis. |
| **M – Distant metastasis** |
| M0 No distant metastasis. |
| M1 Distant metastasis. |
| **TNM staging** |
| T1 T2 T3a T3b T4 |
| N0 and M0 I II IIIA IIIB IIIC |
| N1 and M0 IVA IVA IVA IVA IVA |
| Any N and M1 IVB IVB IVB IVB IVB |

**SUPPLEMENTARY TABLE 2 |** Comparison of serum sphingolipids between cirrhosis and HCC or AFP-negative HCC patients

| **Sphingolipid** | **Cirrhosis (N=104)** | **HCC (N=72)** | ***Z^†^*** | ***P^‡^*** | **AFP-negative HCC (N=24)** | ***Z^†^*** | ***P^‡^*** |
| --- | --- | --- | --- | --- | --- | --- | --- |
| **C**er(d18:1/**10:0)** | 205.13(205.1-211.03) | 205.14(205.11-207.34) | -2.21 | 0.03 | 205.15(205.11-205.33) | -1.92 | 0.06 |
| **C**er(d18:1/**12:0)** | 168.59(168.43-172.46) | 168.67(168.45-172.91) | -2.71 | 0.01 | 168.63(168.45-170.17) | -1.03 | 0.30 |
| **C**er(d18:1/**14:0)** | 139.66(91.51-817.98) | 145.22(77.38-253.62) | -0.46 | 0.65 | 145.07(107.37-213.05) | -0.09 | 0.93 |
| **C**er(d18:1/**16:0)** | 884.88(329.09-4652.65) | 786.3(117.23-3007.8) | -1.77 | 0.08 | 733.73(323.73-3007.8) | -2.42 | 0.02 |
| **C**er(d18:1/**18:0)** | 117.14(13.65-1807.99) | 142.63(7.41-1411.56) | -1.5 | 0.13 | 121.59(38.38-1411.56) | -0.26 | 0.79 |
| **C**er(d18:1/**18:1)** | 103.42(9.06-1524.57) | 138.05(1.92-1507.33) | -2.02 | 0.04 | 113.31(20.53-1507.33) | -0.84 | 0.40 |
| **C**er(d18:1/**20:0)** | 18.08(0-2941.1) | 69.9(0-1617.48) | -1.97 | 0.05 | 26.94(0-1617.48) | -0.41 | 0.69 |
| **C**er(d18:1/**22:0)** | 3682.83(1057.92-22532.13) | 3522.52(0-18915.22) | -1.99 | 0.05 | 3502.13(1497.96-18915.22) | -0.7 | 0.49 |
| **C**er(d18:1/**24:0)** | 10096.32(1433.58-34343.97) | 10146.95(245.34-89430.63) | -0.17 | 0.87 | 10451.39(3735.8-89430.63) | -0.41 | 0.68 |
| **C**er(d18:1/**24:1)** | 24344.61(3001.57-87356.87) | 25066.25(0-227631.52) | -0.06 | 0.95 | 25929.75(8299.57-227631.52) | -0.55 | 0.58 |
| **C**er(d18:1/**26:0)** | 384.8(123.07-1745.91) | 655.54(70.75-4583.91) | -3.78 | <0.01 | 594.05(179.54-4583.91) | -2.73 | 0.01 |
| **C**er(d18:1/**26:1)** | 904.13(215.88-4666.05) | 1536.52(85.84-12766.88) | -3.72 | <0.01 | 1436.24(356.92-12766.88) | -2.59 | 0.01 |
| **C**er(d18:1/**12:1)-1-P** | 82.9(79.03-235.91) | 84.4(79.32-631.88) | -1.82 | 0.07 | 85.79(80.26-107.17) | -2.75 | <0.01 |
| **C**er(d18:1/**14:1)-1-P** | 59.72(59.06-64.8) | 59.94(59.11-64.41) | -1.58 | 0.12 | 60.02(59.11-63.16) | -1.34 | 0.18 |
| **C**er(d18:1/**14:0)-1-Pa** | 60.38(59.22-71.09) | 60.49(59.36-70.36) | -1.23 | 0.22 | 60.35(59.36-65.08) | -0.95 | 0.34 |
| **C**er(d18:1/**14:0)-1-Pb** | 85.55(61.91-390.16) | 93.55(59.73-180.03) | -2.56 | 0.01 | 94.49(65.78-180.03) | -1.69 | 0.09 |
| **C**er(d18:1/**16:0)-1-P** | 71.9(59.57-351.01) | 75.88(60.06-185.03) | -1.59 | 0.11 | 72.57(60.51-133.69) | -0.08 | 0.93 |
| **C**er(d18:1/**16:1)-1-P** | 63.18(59.2-243.99) | 67.34(59.34-147.75) | -2.48 | 0.01 | 68.68(59.37-124.7) | -2.42 | 0.02 |
| **C**er(d18:1/**18:0)-1-P** | 4210.6(1745.57-32192.67) | 4703.36(125.34-17601.75) | -0.99 | 0.32 | 4548.47(2058.24-17601.75) | -0.47 | 0.64 |
| **C**er(d18:1/**18:1)-1-P** | 468.57(165.4-7878.09) | 602.77(61.74-2539.12) | -1.88 | 0.06 | 576.78(192.64-2216.08) | -0.85 | 0.40 |
| **C**er(d18:1/**20:0)-1-P** | 251.65(104.5-3622.69) | 283.48(60.51-1188.99) | -0.81 | 0.42 | 233.88(90.98-1079.78) | -0.16 | 0.87 |
| **C**er(d18:1/**20:1)-1-P** | 73.62(60.55-1511.68) | 87.69(60.13-216.47) | -3.08 | <0.01 | 86.3(62.08-142.09) | -1.75 | 0.08 |
| **C**er(d18:1/**22:0)-1-P** | 72.35(60.55-166.01) | 77.23(60.85-337.1) | -2.06 | 0.04 | 76.34(60.85-205.76) | -0.93 | 0.35 |
| **C**er(d18:1/**22:1)-1-P** | 60.16(59-67.61) | 60.4(59.22-67.31) | -1.56 | 0.12 | 60.6(59.3-67.31) | -2.34 | 0.02 |
| **C**er(d18:1/**26:0)-1-P** | 516.87(189.74-2958.24) | 658.34(64.81-3033.7) | -2.86 | 0.01 | 572.72(287.9-1555.19) | -0.88 | 0.38 |
| **C**er(d18:1/**26:1)-1-P** | 474.38(151.7-2566.19) | 589.55(66.18-2582.93) | -2.76 | 0.01 | 502(257.06-1374.65) | -1.1 | 0.27 |
| **C**er(d18:1/**28:0)-1-P** | 212.44(101.3-1283.09) | 256.23(62.89-1975.61) | -2.44 | 0.02 | 229.2(103.68-765.68) | -0.51 | 0.61 |
| **C**er(d18:1/**28:1)-1-P** | 363.51(126.69-1637.51) | 486.64(61.27-2053.08) | -3.82 | <0.01 | 435.63(206.12-1028.54) | -1.54 | 0.12 |
| HexCer (d18:1/**8:0)** | 184.27(184.22-186) | 184.32(184.23-201.05) | -3.33 | <0.01 | 184.31(184.23-185.5) | -1.9 | 0.06 |
| HexCer (d18:1/**12:0)** | 63.33(63.09-78.46) | 63.37(63.11-68.68) | -0.23 | 0.82 | 63.25(63.13-68.22) | -1.33 | 0.18 |
| HexCer (d18:1/**14:0)** | 210.37(206.4-239.56) | 211.12(206.86-257.09) | -1.49 | 0.14 | 209.48(207.7-213.95) | -1.54 | 0.13 |
| HexCer (d18:1/**14:1)** | 213.03(206.88-267.83) | 214.9(208.92-314.7) | -2.53 | 0.01 | 212.79(209.49-222.56) | -0.35 | 0.73 |
| HexCer (d18:1/**16:0)** | 17010.25(6981.37-82297.98) | 15565.12(138.86-63466.28) | -1.39 | 0.16 | 12960.89(7318.84-30826.31) | -2.2 | 0.03 |
| HexCer (d18:1/**16:1)** | 563.6(290.14-4207.56) | 602.88(206.84-1909.08) | -0.67 | 0.5 | 542.47(365.41-876.1) | -0.67 | 0.51 |
| HexCer (d18:1/**18:0)** | 343.17(165.47-1298.07) | 322.45(84.18-3464.06) | -2.42 | 0.02 | 313.28(173.9-456.75) | -2.39 | 0.02 |
| HexCer(d18:1/**18:1)** | 474.92(176.39-2092.78) | 395.75(66.67-6444.01) | -2.7 | 0.01 | 395.75(210.57-685.74) | -2.45 | 0.01 |
| HexCer (d18:1/**20:0)** | 188.95(108.93-519.85) | 203.52(69.61-1116.98) | -0.35 | 0.73 | 201.99(112.18-275.81) | -0.23 | 0.82 |
| HexCer (d18:1/**20:1)** | 383.06(167.67-1052.18) | 391.52(80.54-2762.2) | -0.34 | 0.74 | 381.46(192.99-597.97) | -0.32 | 0.75 |
| HexCer (d18:1/**22:0)** | 1290.44(389.18-4577.75) | 1372.84(66.04-5941.23) | -0.37 | 0.71 | 1753.82(408.53-2798) | -0.97 | 0.33 |
| HexCer(d18:1/**22:1)** | 168.08(74.73-1334.6) | 190.2(61.3-775.58) | -0.55 | 0.59 | 191.94(79.25-593.39) | -0.23 | 0.82 |
| HexCer (d18:1/**24:0)** | 3495.17(857.77-9131.55) | 3176.82(81.22-12081.43) | -0.34 | 0.73 | 3625.48(1292.38-10656.07) | -0.08 | 0.94 |
| HexCer (d18:1/**24:1)** | 61707.52(15222.56-151402.39) | 46412.97(0-144596.58) | -3.02 | <0.01 | 47517.08(27581.79-144596.58) | -1.78 | 0.08 |
| **dhS**ph (d18:1/**18:0)** | 24.14(18.79-81.61) | 29.56(19.18-176.67) | -4.89 | <0.01 | 29.75(19.42-128.31) | -3.04 | <0.01 |
| **dhSph**(d18:1/**18:0)-1-P** | 56.03(55.89-58.98) | 56.46(55.93-60.01) | -5.61 | <0.01 | 56.32(56-58.02) | -3.56 | <0.01 |
| dhCer(d18:0/**18:0)** | 60.32(59.11-81.33) | 60.69(59.06-71.91) | -1.61 | 0.11 | 60.76(59.06-71.91) | -1.12 | 0.26 |
| dhCer (d18:0/**18:1)** | 62.32(59.33-153.02) | 62.08(59.12-97.34) | -0.1 | 0.92 | 61.18(59.12-79.22) | -1.06 | 0.29 |
| dhCer (d18:0/**20:0)** | 61.15(59.19-108.81) | 62.4(59.45-83.77) | -1.24 | 0.22 | 61.5(59.45-83.77) | -0.17 | 0.87 |
| **dhC**er(d18:0/**24:0)** | 614.63(156.08-4343.21) | 531.45(137.82-1955.56) | -1.26 | 0.21 | 478.64(252.07-1184.92) | -2.01 | 0.04 |
| dhCer (d18:0/**24:1)** | 1415.43(172.52-6220.29) | 1559.88(78.06-16626.77) | -0.9 | 0.37 | 61.55(59.3-69.69) | -1.07 | 0.28 |
| dhCer(d18:0/**16:0)-1-P** | 195.26(69.1-1880.24) | 172.3(63.52-641.53) | -1.98 | 0.05 | 197.17(97.45-620.76) | -0.58 | 0.56 |
| dhCer(d18:0/**16:1)-1-P** | 117.98(68.15-980.21) | 140.1(59.71-802.81) | -1.63 | 0.1 | 144.1(86.85-802.81) | -2.27 | 0.02 |
| dhCer(d18:0/**18:0)-1-P** | 137.43(75.08-970.98) | 157.01(62.37-504.63) | -0.9 | 0.37 | 136.37(69.74-287.56) | -0.31 | 0.76 |
| dhCer(d18:0/**18:1)-1-P** | 67.26(61.24-768.87) | 68.84(59.99-96.73) | -0.93 | 0.35 | 69.8(60.26-96.73) | -0.76 | 0.45 |
| dhCer(d18:0/**24:1)-1-P** | 59.63(59.08-72.41) | 61(59.08-69.69) | -4.94 | <0.01 | 61.55(59.3-69.69) | -3.93 | <0.01 |
| HexSph(d18:1) | 45.1(44.64-76.62) | 46.02(44.71-225.41) | -4.85 | <0.01 | 46.31(44.71-105.12) | -2.92 | <0.01 |
| Sphingosine(d18:1)**-1-P** | 55.96(55.89-59.12) | 57.13(55.89-67.94) | -7.83 | <0.01 | 56.94(55.89-64.44) | -4.41 | <0.01 |
| Sphingosine (d18:1) | 69.04(59.16-198.47) | 80.84(56.2-241.79) | -4.17 | <0.01 | 73.85(57.44-183.81) | -1.76 | 0.08 |

*Unit of sphingolipid levels: pmol/0.1 mL serum.*

*Data are expressed as median(range).*

*P^†^ indicates the comparison between cirrhosis and HCC.*

*P^‡^ indicates the comparison between cirrhosis and AFP-negative HCC.*

*P<0.05 is considered to be statistically significant.*
